# Heterologous expression of linoleic acid isomerase from *Propionibacterium acnes* and anti-proliferative activity of recombinant *trans-*10, *cis-*12 conjugated linoleic acid

**DOI:** 10.1099/mic.0.2006/001966-0

**Published:** 2007-08

**Authors:** Eva Rosberg-Cody, Mark C. Johnson, Gerald F. Fitzgerald, Paul R. Ross, Catherine Stanton

**Affiliations:** 1 Teagasc, Moorepark Food Research, Biotechnology Centre, Fermoy, Co. Cork, Ireland; 2 Microbiology Department, University College Cork, Ireland; 3 Alimentary Pharmabiotic Centre, Cork, Ireland

## Abstract

The linoleic acid isomerase enzyme from *Propionibacterium acnes* responsible for bioconversion of linoleic acid to *trans-*10, *cis-*12 conjugated linoleic acid (*t*10, *c*12 CLA) was cloned and overexpressed in *Lactococcus lactis* and *Escherichia coli*, resulting in between 30 and 50 % conversion rates of the substrate linoleic acid to *t*10, *c*12 CLA. The anti-proliferative activities of the fatty acids produced following isomerization of linoleic acid by *L. lactis* and *E. coli* were assessed using the human SW480 colon cancer cell line. Fatty acids generated from both *L. lactis* and *E. coli* contained a mixture of linoleic acid and *t*10, *c*12 CLA at a ratio of ∼1.35 : 1. Following 5 days of incubation of SW480 cells with 5–20 μg ml^−1^ (17.8–71.3 μM) of the *t*10, *c*12 CLA, there was a significant (*P*<0.001) reduction in growth of the SW480 cancer cells compared with the linoleic acid control. Cell viability after treatment with the highest concentration (20 μg ml^−1^) of the *t*10, *c*12 CLA was reduced to 7.9 % (*L. lactis* CLA) and 19.6 % (*E. coli* CLA), compared with 95.4 % (control linoleic acid) and 31.7 % (pure *t*10, *c*12 CLA). In conclusion, this is believed to represent the first report in which recombinant strains are capable of producing CLA with an anti-proliferative potential.

## INTRODUCTION

Conjugated linoleic acids (CLAs) are a family of positional and geometric isomers of linoleic acid with conjugated double bonds. Biological activities associated with the *cis-*9, *trans-*11 CLA (*c*9, *t*11 CLA) and the *trans-*10, *cis-*12 CLA (*t*10, *c*12 CLA) isomers include anti-cancer, anti-atherosclerotic, anti-diabetic and immune-enhancing properties, and positive effects on body composition and bone formation (Belury, 2002; Pariza *et al.*, 1999, 2000). A number of studies have shown that the *t*10, *c*12 CLA isomer is the most potent isomer for prevention of cell proliferation and induction of apoptosis in cancer cells (Cho *et al.*, 2005, 2006; Lee *et al.*, 2006; Kim *et al.*, 2002a; Ochoa *et al.*, 2004). Furthermore, this isomer has the ability to alter body composition by reducing the fat content and increasing the lean body tissue in animal models, and also in some studies with humans (Smedman & Vessby, 2001; Blankson *et al.*, 2000; Thom *et al.*, 2001).

*c*9, *t*11 CLA is formed as an intermediate during biohydrogenation of linoleic acid by rumen bacteria, and consequently natural sources of this isomer are milk and fats from ruminants. The main CLA isomer in milk fat is *c*9, *t*11 CLA, which accounts for 80–90 % of total milk fat CLA, whereas the *t*10, *c*12 CLA isomer is only present at about 1 % (Jensen, 2002). A range of cultures with the ability to convert linoleic acid to *c*9, *t*11 CLA are known, in addition to the rumen microflora. We have previously shown that some human-derived *Bifidobacterium* species can produce CLA (Coakley *et al.*, 2003; Rosberg-Cody *et al.*, 2004). The predominant isomer formed after 48–72 h incubation with 0.5 mg linoleic acid ml^−1^ is *c*9, *t*11 CLA, with some of this isomer further converted to the *t*9, *t*11 CLA isomer (Coakley *et al.*, 2006). Other species reported to biosynthesize CLA, mainly the *c*9, *t*11 CLA isomer, are propionibacteria used as dairy starter cultures (Jiang *et al.*, 1998), strains of the intestinal flora of rats (Chin *et al.*, 1994), *Lactobacillus* (Lin *et al*., 1999), *Corynebacterium* (Fairbank *et al.*, 1988, 1989), *Lactococcus* (Kim & Liu, 2002) and some common bacterial lung pathogens (Jack *et al.*, 1994). The only known species which can perform a *c*9 to *t*10 isomerization are *Propionibacterium acnes* isolated from mouse caecum (Verhulst *et al.*, 1987) and the rumen bacterium *Megasphaera elsdenii* (Kim *et al.* 2000, 2002b). *P. acnes* has been shown to convert ∼85 % of linoleic acid into *t*10, *c*12 CLA after 24 h of incubation and ∼10 % into *t*10 octadecenoic acid (Verhulst *et al.*, 1987). Since both reaction products have proved to be derived from linoleic acid, the results indicate that the *c*9 double bond of linoleic acid is first isomerized into a *t*10 double bond, partly followed by reduction of the *c*12 double bond (Verhulst *et al.*, 1987). The *t*10, *c*12 CLA could be formed from linoleic acid alone by *P. acnes* and *M. elsdenii*, and not from linolenic acid, as with the *c*9, *t*11 CLA isomer (Verhulst *et al.*, 1987; Kim *et al.*, 2000).

The mechanisms by which CLA exerts all its physiological effects are not yet fully understood, but at least two different pathways have been proposed. The first suggests that CLA reduces the arachidonate pool, leading to a reduced production of downstream eicosanoid products, which modulates cytokine production involved in inflammation and cancer. The other model includes the regulation of expression of genes known to control the cell cycle and apoptosis, but also lipid oxidation, adipocyte differentiation, energy balance and atherogenesis (Belury, 2002; Pariza *et al.*, 2000). Recently, it has been proposed that different CLA isomers produce different effects. The *t*10, *c*12 CLA isomer seems to work preferentially through modulation of apoptosis and cell cycle control, while *c*9, *t*11 CLA appears to affect arachidonic acid metabolism. The *t*10, *c*12 CLA isomer has been shown to control the cell cycle and induce apoptosis by decreasing bcl-2 gene expression and increasing p21^WAF1/Cip1^ mRNA levels in prostate cancer cells (Ochoa *et al.*, 2004). It has been shown to activate caspase-3, and -9, translocate the anti-apoptotic protein Bax into the mitochondrial membrane and cleave the anti-apoptotic protein Bid in rat hepatoma cells (Yamasaki *et al.*, 2005), along with induction of caspase-dependent apoptosis in MIP-101 and PC-3 cells (Palombo *et al.*, 2002). This isomer has also been reported to decrease cell growth due to a decrease in the secretion of insulin-like growth factor II (IGF-II) in Caco-2 colon cancer cells (Kim *et al.*, 2002a), and to cause a decrease of expression of the oncogene ErbB3 in HT-29 human colon cancer cells (Cho *et al.*, 2005). Lee *et al.* (2006) have reported that *t*10, *c*12 CLA induces pro-apoptotic and anti-tumorigenic NAG-1 in human colorectal cancer cells. Moreover, *t*10, *c*12 CLA induces p53, p21 and p27, and reduces the levels of cyclins D1 and E and hyperphosphorylated retinoblastoma protein (required for G1 to S phase transition) in human breast cancer cells (Kemp et *al*., 2003). Cho *et al.* (2006) have reported the induction by *t*10, *c*12 CLA of G1 cell cycle arrest through increased expression of p21, as well as decreased CDK2 activity, decreased levels of hyperphosphorylated retinoblastoma (Rb) protein and increased hypophosphorylated Rb protein in HT-29 colon cancer cells. However, an integrated model of the mechanism of action of *t*10, *c*12 CLA has not been developed to date.

The aims of this study were to clone and overexpress the linoleic acid isomerase capable of producing *t*10, *c*12 CLA from *P. acnes* (Hornung *et al.*, 2005) in *Lactococcus lactis* and *Escherichia coli*, and to investigate the anti-proliferative activity of the generated *t*10, *c*12 CLA using the human SW480 colon cancer cell line.

## METHODS

### Cultures and media.

*L. lactis* NZ9800 (an *L. lactis* NZ9700 derivative which, due to a deletion in the *nis*A gene, does not produce nisin, and which contains the *nis*RK signal transduction genes integrated on the chromosome) was cultured at 30 °C in M17 (Difco) broth and/or agar containing glucose (0.5 %, w/v). *L. lactis* carrying the plasmid pNZ44 was routinely cultured in the presence of chloramphenicol (5 μg ml^−1^) as a selective marker. *E. coli* DH5*α* (TOP 10, Invitrogen) harbouring the plasmid pNZ44 was cultured in Luria–Bertani (LB) media supplemented with chloramphenicol (20 μg ml^−1^) with shaking at 37 °C.

Human colon cancer cells were obtained from the American Type Culture Collection (ATCC). The SW480 cell line was originally established from a primary adenocarcinoma of the colon (Leibovitz *et al.*, 1976). The cell line is somewhat dedifferentiated, lacking many of the phenotypic markers of normal colon epithelial cells. SW480 cells do retain an epithelial-like morphology, with regular polygonal cells predominating in culture, with some round cells. The *t*10, *c*12 CLA isomer (>98 % purity) was obtained from Matreya and linoleic acid was purchased from Sigma Aldrich. Cell culture media and supplements were purchased from Sigma, unless otherwise stated. SW480 cells were maintained in Dulbecco's Minimum Essential Medium (DMEM) supplemented with 5 % (v/v) fetal bovine serum, 0.2 mM l-glutamine, 1 mM HEPES, and 1 U ml^−1^ penicillin and streptomycin. SW480 cells were grown in 75 ml flasks and maintained at 37 °C in a humidified atmosphere at pH 7.2–7.4 with a flow of 95 % air/5 % CO_2_, as required.

### DNA manipulation.

Two oligonucleotide primers were designed to amplify the complete linoleic acid isomerase (coPAI) for production of *t*10, *c*12 CLA from the original construct pC33.1-coPAI (linoleic acid isomerase gene in a plant vector; BASF). The forward primer, designated ERcoPAI1, contained a *Pst*I restriction site and a ribosome-binding site (RBS), four extra bases at the 5′ end and seven extra bases between the RBS and the gene start: 5′-aaaactgcagaggaggaaaaaaa**ATGGGTTCCATTTCCAAGGA**-3′, where bold capitals represent part of the DNA sequence and small capitals represent artificial sequence (e.g. restriction sites and added bases). The reverse primer, designated ERcoPAI2, contained a *Kpn*I restriction site and three extra bases at the 5′ end: 5′-cggggtacc**TCACACGAAGAACCGCGTCA**-3′. The 1278 bp coPAI gene was amplified in an Eppendorf Mastercycler Gradient with High Fidelity Expand polymerase, as described by the supplier (Roche Diagnostics) using 200 ng plasmid DNA (pC33.1-coPAI) as template. PCR reactions were performed in a total volume of 50 μl containing 1 μl of each primer, 3 mM MgCl_2,_ 5 μl 10× Expand buffer, 1 μl dNTPs and 0.75 μl Expand polymerase. PCR conditions were as follows: one cycle of 2 min denaturation (94 °C), 10 cycles of 15 s denaturation (94 °C), 30 s annealing (55 °C), 2 min elongation (72 °C), followed by 20 cycles of 15 s (94 °C), 30 s (55 °C), 2 min+5 s per cycle (72 °C), and finally one 7 min cycle at 72 °C. The PCR reaction mixture was analysed in a 1 % (w/v) agarose gel to visualize the resulting PCR fragment.

**Fig. 1. f1:**
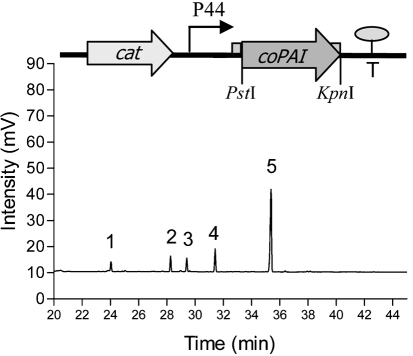
The pEV01 construct (above) and a representative GLC chromatogram (below) of supernatant following 72 h incubation of *L. lactis* pEV01 in media containing 0.5 mg linoleic acid ml^−1^. Peaks: 1, C16 : 0 (palmitic acid); 2, C18 : 0 (stearic acid); 3, C18 : 1 (oleic acid); 4, C18 : 2 (linoleic acid); 5, C18 : 2 (*t*10, *c*12 CLA).

The Qiagen Plasmid Mini kit was used to isolate plasmid DNA from *E. coli* and *L. lactis* NZ9800, with one minor modification for *L. lactis*, i.e. 40 mg lysozyme ml^−1^ was added to buffer P1 and incubated for 20 min at 37 °C. PCR products were purified using a Qiaquick PCR Purification kit (Qiagen). The two plasmids pNZ8048 (nisin-inducible plasmid containing P*nis*A promoter, initially used for cloning of the coPAI gene) and pNZ44 (a derivative of pNZ8048 in which the P*nis*A promoter is replaced by P44, a constitutive *L. lactis* chromosomal promoter), and the coPAI gene fragment, were restricted with *Pst*I and *Kpn*I, followed by a ligation reaction at 15 °C with T4 DNA ligase, as described by the supplier (New England Biolabs), resulting in the construct pEV01, shown in Fig. 1. Recombinant plasmids were double-digested with the same enzymes to verify the correct clone and then electroporated into *L. lactis* NZ9800. Electrocompetent *L. lactis* was prepared and transformed according to the method described by de Ruyter *et al.* (1996). Chemically competent *E. coli* DH5*α* (TOP 10) was purchased from Invitrogen. The authenticity of the clones was verified by sequencing (MWG-Biotech) and sequence analysis was performed using dnastar software.

### Screening for CLA production.

The *L. lactis* pEV01 and *E. coli* pEV01 clones were tested for their ability to convert free linoleic acid (0.1–0.5 mg ml^−1^) to *t*10, *c*12 CLA as follows. Inoculum (1 %) of an overnight culture was transferred to 10 ml of the appropriate broth and incubated until the culture reached OD_600_ ∼0.5. Then, linoleic acid (0.1–0.5 mg ml^−1^) (added from a 30 mg ml^−1^ stock solution, sterile-filtered through a 0.45 μm filter, Sarstedt) was added to cultures, followed by further incubation to 72 h. Following incubation, the culture was centrifuged (4345 ***g***) for 20 min and fatty acids were extracted from the supernatant and cell pellets, followed by methylation and analysis by GLC, as described by Coakley *et al.* (2003). All conversion rates (%) were related to the amount of linoleic acid that was recovered and extracted from the media following incubation without culture for the same time as with culture, which represents 100 % of available linoleic acid. All experiments were done at least in triplicate.

### Preparation of fatty acids.

The *E. coli* and *L. lactis* pEV01 clones, as well as *L. lactis* and *E .coli* vector controls (empty pNZ44) were inoculated (1 % overnight culture) into 500 ml of the respective media and grown to OD_600_ ∼0.5, after which linoleic acid (0.5 mg ml^−1^) was added and incubation continued to 72 h. A linoleic acid control consisting of medium containing linoleic acid (0.5 mg ml^−1^) but without culture was also prepared and incubated at 37 °C for 72 h. After 72 h, fatty acids (fermented oils) were co-extracted from the supernatant and the bacterial pellet, as described for the supernatant (Coakley *et al.*, 2003), but with the difference that the supernatant was not separated from the growing culture by centrifugation prior to fatty acid extraction. Control samples from fermentations were also prepared, methylated and analysed by GLC in triplicate to calculate the amounts of fatty acids and the CLA : linoleic acid ratio for the samples.

### Anti-proliferative activity of fatty acids on human SW480 colon cancer cells.

To examine the anti-proliferative activity of the fatty acids extracted following fermentation of cultures of *L. lactis* and *E. coli* pEV01 in the respective media containing linoleic acid (0.5 mg ml^−1^), SW480 human colon cancer cells were cultured in the presence of different concentrations of the extracted fatty acids. The fermented oils generated from both *L. lactis* and *E. coli* (pEV01) expressing coPAI contained a mixture of linoleic acid and *t*10, *c*12 CLA at a ratio of ∼1.35 : 1. All samples, except for the pure *t*10, *c*12 CLA standard, contained smaller amounts of palmitic, stearic and oleic acids. *E. coli* fermented oil also contained 1.6 % *t*9, *t*11 CLA (Table 1). Since the recombinantly produced *t*10, *c*12 CLA samples contained even more linoleic acid as well as other fatty acids, all calculated concentrations added to the SW480 cancer cells were based on the amount of *t*10, *c*12 CLA present in the samples (or linoleic acid in the control sample, and the fermented oil samples from the *L. lactis* and *E. coli* vector controls). Initially, 1×10^4^ cells were seeded per well in 96-well plates and cultured for 24 h at 37 °C to allow the cells to adhere to the surface prior to treatment with 5–20 μg (ml medium)^−1^ *t*10, *c*12 CLA (bacterial *t*10, *c*12 CLA/linoleic acid mix and pure Matreya standard in ethanol) and 5–25 μg (ml medium)^−1^ linoleic acid (control fatty acid extracted from broth). All fatty acids were sterile-filtered through a 0.2 μm Millex FG filter unit (Millipore) for non-aqueous solvents. Control flasks were supplemented with ethanol to a final concentration of 0.1 % (v/v). Following incubation for 5 days, cell viability was measured and relative cell numbers were determined using the MTS method (Promega), a colorimetric method for determining the number of viable cells in proliferation or cytotoxicity assays, subsequent to incubation with MTS tetrazolium compound. Following incubation with MTS for 2 h, *A*_492_ was recorded with a 96-well plate reader. Cell viability (%) after treatment was expressed relative to the ethanol control (100 %). Three independent experiments were performed on separate occasions, each time in triplicate for each fatty acid/fermented oil treatment of SW480. Student's *t* test was used to determine significant differences between treatments (*P*<0.001).

## RESULTS

### Sequence analysis

The 1278 bp gene from *P. acnes* (Hornung *et al.*, 2005) encodes a linoleic acid isomerase protein of 425 aa. The molecular mass of the isomerase is 49 077 Da. Comparison with sequences in the database revealed that the cloned isomerase protein showed significant homology with flavin-containing amino oxidases over most of the sequence (approx. amino acids 25–400; NCBI Conserved Domain Database; http://www.ncbi.nlm.nih.gov/Structure/cdd/cdd.shtml). The isomerase showed 96 % identity to a putative amino oxidase from *P. acnes* (GenBank accession no. Q6A8X5_PROAC; EXPASY/UniProtKB database), but only 26 % identity to the next best match, a protein from the plant *Oryza sativa* (japonica cultivar group; accession no. Q7XR12_ORYSA; EXPASY/UniProtKB database) (http://www.expasy.ch/cgi-bin/blast.pl) spanning amino acids 145–423. The aligned region in these proteins includes a flavin binding site. The flavin-containing amine oxidase family also contains phytoene hydrogenases and related enzymes. An NAD/FAD binding domain located in the region between amino acid residues 10 and 39 was identified by the Prosite database (http://www.expasy.org/prosite/). The crystal structure of the isomerase protein PAI (*P. acnes* isomerase) has recently been determined (Liavonchanka *et al.*, 2006), which has confirmed the protein to be an FAD-containing monomer consisting of three intricately connected domains. Structural comparisons also show that the closest homologues of the isomerase protein domain 1, identified by the DALI program (www.ebi.ac.uk/dali) are FAD-containing oxidoreductases and isomerases, such as yeast polyamine oxidases. No structural homologues of PAI domains 2 and 3 have been found (DALI database) (Liavonchanka *et al.*, 2006). The isomerase protein is soluble (http://www.psort.org/psortb/) and the predicted location of the protein is cytoplasmic (http://bp.nuap.nagoya-u.ac.jp/sosui/sosuimenu0E.html).

### Bioconversion of linoleic acid

**Fig. 2. f2:**
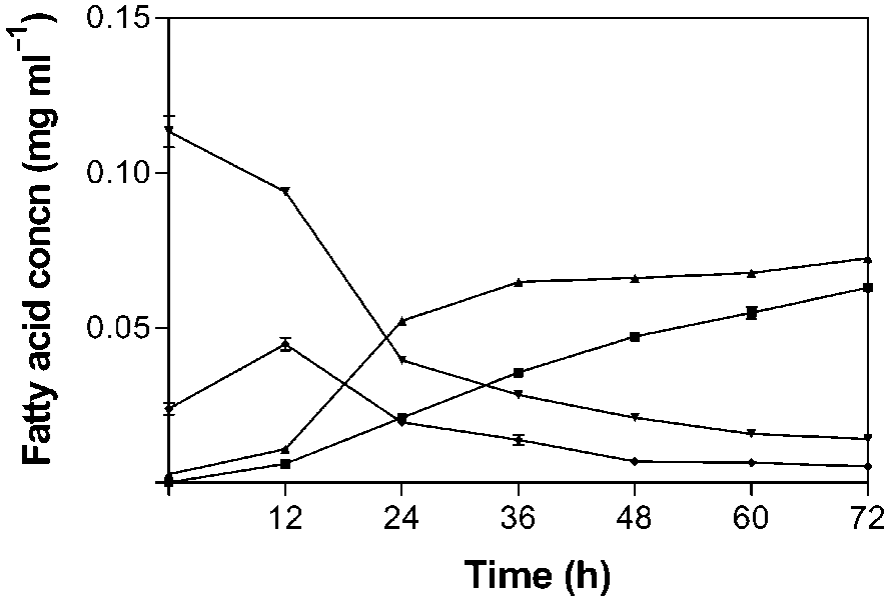
*t*10, *c*12 CLA production versus linoleic acid usage and accumulation of the fatty acids in the cell pellets by *L. lactis* pEV01 incubated with 0.2 mg linoleic acid ml^−1^ for 72 h. The culture was incubated in linoleic acid at OD_600_ 0.5. Samples were analysed in triplicate. ▪, *t*10, *c*12 CLA supernatant; ▾, linoleic acid supernatant; ▴, *t*10, *c*12 CLA bacterial pellets; ⧫, linoleic acid bacterial pellets.

*L. lactis* carrying the construct pEV01 was shown to convert free linoleic acid into *t*10, *c*12 CLA, while the control *L. lactis* culture containing the vector pNZ44 alone produced no CLA isomers. *L. lactis* pEV01 converted >50 % of the free linoleic acid to *t*10, *c*12 CLA (Figs 1 and 2). Interestingly, *L. lactis* pEV01 accumulated a large amount of *t*10, *c*12 CLA in the cell pellets. After 72 h incubation, up to 60 % of total *t*10, *c*12 CLA was recovered in the bacterial membranes. Nearly all the *t*10, *c*12 CLA that was recovered in the membranes was already incorporated after half the incubation time (36 h). At this time point, the concentration of *t*10, *c*12 CLA in the supernatant was only about half of the final concentration (Fig. 2). Given that *L. lactis* did not grow well when initially incubated in the presence of linoleic acid (0.4–0.5 mg ml^−1^), the fatty acid was instead added to the growing culture at OD_600_ 0.5. At this growth stage, the culture still showed sensitivity to linoleic acid, resulting in a reduced growth rate at this linoleic acid concentration. Greater rates of conversion to *t*10, *c*12 CLA were observed at lower concentrations of free linoleic acid (0.1 and 0.2 mg ml^−1^). *E. coli* cells carrying the construct pEV01 converted about 40 % of linoleic acid after 72 h incubation in the presence of the fatty acid (0.5 mg ml^−1^), whereas *E. coli* pNZ44 (vector control) did not produce any CLA. Compared with *L. lactis* pEV01, *E. coli* pEV01 accumulated much less CLA in the membranes: only about 10 % of total *t*10, *c*12 CLA.

### Anti-proliferative activity of microbially generated fatty acids on human SW480 colon cancer cells

**Fig. 3. f3:**
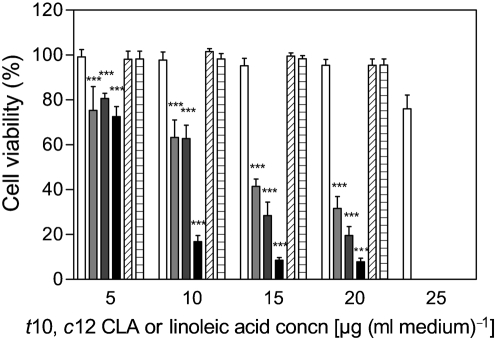
Cell viability of SW480 cells after 5 days of incubation with 5–20 μg (ml medium)^−1^ *t*10, *c*12 CLA (fermented oils from CLA-producing *L. lactis* and *E. coli* or pure *t*10, *c*12 CLA) or 5–25 μg (ml medium)^−1^ linoleic acid (control linoleic acid extracted from broth and fermented oils of *L. lactis* and *E. coli* vector controls). Data represent cell viability expressed as a percentage of the ethanol control, which was taken as 100 %. White bars, control linoleic acid (extracted from broth after 72 h incubation); light-grey bars, *E. coli* pEV01 fermented oil; dark-grey bars, pure *t*10, *c*12 CLA standard (Matreya); black bars, *L. lactis* pEV01 fermented oil; diagonal-hatched bars, *L. lactis* vector control fermented oil; horizontal-hatched bars, *E. coli* vector control fermented oil. Since the recombinantly produced *t*10, *c*12 CLA samples also contained other fatty acids, all calculated concentrations were based on the amount of *t*10, *c*12 CLA (or linoleic acid in the control samples) present in the samples. Three independent experiments were performed on separate occasions and each time in triplicate for each fatty acid treatment, and Student's *t* test was used to determine significant differences between treatments. Error bars represent sd. ***Values significantly different (*P*<0.001) from that of the control (linoleic acid extracted from broth).

To investigate the anti-proliferative effects of fatty acids produced following isomerization of linoleic acid by *L. lactis* pEV01 and *E. coli* pEV01, human colon cancer cells (SW480) were cultured in the presence of the extracted fermented oils (consisting of a mixture of linoleic acid and *t*10, *c*12 CLA at a ratio of ∼1.35 : 1, and smaller amounts of other fatty acids; Table 1). Controls of linoleic acid extracted from LB broth after 72 h incubation at 37 °C and of the pure synthetic *t*10, *c*12 CLA standard, but also controls of fermented oils from *L. lactis* and *E. coli* transformed with the empty vector, were inoculated with SW480 cells. Since linoleic acid has been shown to have an anti-proliferative effect on SW480 cancer cells at higher concentrations [42.8 μg (ml medium)^−1^; 152.5 μM], and a slightly proliferative effect at a lower concentration [16.9 μg (ml medium)^−1^; 60.2 μM] (Miller *et al.,* 2003), concentrations of *t*10, *c*12 CLA (microbially generated fatty acid samples) between 5 and 20 μg (ml medium)^−1^ [equivalent to 6.7–27 μg linoleic acid (ml medium)^−1^ in the same fatty acid sample] were chosen so as not to exceed the threshold concentration at which linoleic acid inhibits cell growth. Therefore, the highest linoleic acid concentration added to the cancer cells was 25 μg (ml medium)^−1^ from the control samples, to examine the effect of linoleic acid alone, given that the fermented oil samples contained approximately this concentration of linoleic acid together with 20 μg (ml medium)^−1^ *t*10, *c*12 CLA. Following 5 days of incubation with *t*10, *c*12 CLA between 5 and 20 μg ml^−1^, there was a significant (*P*<0.001) reduction in growth of the SW480 cancer cells compared with control linoleic acid at all concentrations. Average cell viability following treatment with the highest concentration (20 μg ml^−1^) of *t*10, *c*12 CLA was reduced to 7.9 % (*L. lactis* fermented oil) and 19.6 % (*E. coli* fermented oil), compared with 95.4 % (control linoleic acid+*L. lactis* vector control fermented oils) and 95.6 % (*E. coli* vector control fermented oils) (Fig. 3). Viable cell numbers following treatment with the highest concentration (25 μg ml^−1^) of linoleic acid were decreased to 76 %, which showed that this concentration had a slight anti-proliferative effect on the SW480 cells and therefore must have contributed to the killing effect of the highest concentrations of the fermented oils. All figures are related to ethanol controls (100 % cell viability). Significant differences in cell viability were observed at all concentrations between the control linoleic acid and the *t*10, *c*12 CLA from *L. lactis* and *E. coli* (*P*<0.001). There was no significant difference in cell viability after treatment between *E. coli t*10, *c*12 CLA and the pure *t*10, *c*12 CLA standard at any concentration. However, there was a significant difference in cell viability between treatments with *L. lactis t*10, *c*12 CLA and the pure *t*10, *c*12 CLA standard at concentrations between 10 and 20 μg ml^−1^ (*P*<0.001) (Fig. 3).

## DISCUSSION

We have successfully cloned and overexpressed the *t*10, *c*12 CLA isomerase from *P. acnes* in *L. lactis* and *E. coli.* The recombinant isomerase protein was functionally active, with conversion rates of >50 % of the added linoleic acid to *t*10, *c*12 CLA when expressed in *L. lactis*, and 40 % in *E. coli*. The isomerase protein was also expressed in the probiotic strain *Lactobacillus paracasei* subsp. *paracasei* NFBC 338, which was also capable of converting linoleic acid to *t*10, *c*12 CLA (data not shown). The enzyme has previously been characterized and expressed in bacteria, yeast and tobacco seeds (Rosson *et al.*, 2001; Hornung *et al.*, 2005). However, not many sequences of polyunsaturated fatty acid isomerases are known, and the homology between coPAI and other fatty acid isomerase sequences in the database was not significant. Recently, Liavonchanka *et al.* (2006) have described six crystal structures of PAI, which has revealed a unique gating mechanism for substrate specificity due to conformational changes in a hydrophobic channel toward the active site. The length preference for C18 fatty acids can thus be explained by the geometry of the substrate binding site.

Interestingly, higher conversion rates of linoleic acid to *t*10, *c*12 CLA were achieved with recombinant lactococci than with recombinant *E. coli*, while the former were more sensitive to free linoleic acid. For this reason, the linoleic acid was added to the medium only after the cells had reached OD_600_ ∼0.5. Another notable feature of *L. lactis* CLA production was that approximately half of the produced *t*10, *c*12 CLA was cell associated and could be extracted from pelleted cells. The reason for this is unknown, but could be related to the fact that polyunsaturated fatty acids are antibacterial (Kankaanpää *et al.*, 2001), and the demonstrated incorporation into the membranes might be part of a detoxification mechanism (Jiang *et al.*, 1998) used by *L. lactis.* A positive correlation between the inhibitory activity of a fatty acid and the level to which the fatty acid is incorporated into the cell membrane has been reported (Khulusi *et al.*, 1995). In general, a long-chain fatty acid with a higher degree of unsaturation is more inhibitory than a fatty acid of the same chain length with fewer double bonds. Also, Gram-negative bacteria are less susceptible than Gram-positive bacteria to long-chain unsaturated fatty acids (Jenkins & Courtney, 2003). In particular, linoleic acid is known to exhibit an inhibitory effect towards some bacteria, e.g. *Lactobacillus* (Alonso *et al.*, 2003) and propionic acid bacteria (Jiang *et al.*, 1998).

The anti-proliferative effect of the fatty acids produced by *L. lactis* and *E. coli* on human SW480 cancer cells was confirmed in this study. Cell growth inhibition by *t*10, *c*12 CLA was dose-dependent, with the highest growth inhibitory effect at concentrations of 20 μg ml^−1^ *t*10, *c*12 CLA. Most effective was *L. lactis* fermented oil, which left only 8 % viable cells (compared with the ethanol control). *E. coli* fermented oil caused a reduction of viable cell numbers to 20 %. Linoleic acid alone also showed a slight anti-proliferative effect on the cancer cells following incubation with media containing 25 μg ml^−1^, and therefore must have contributed to the killing effect of the highest concentrations of the fermented oils.

**Fig. 4. f4:**
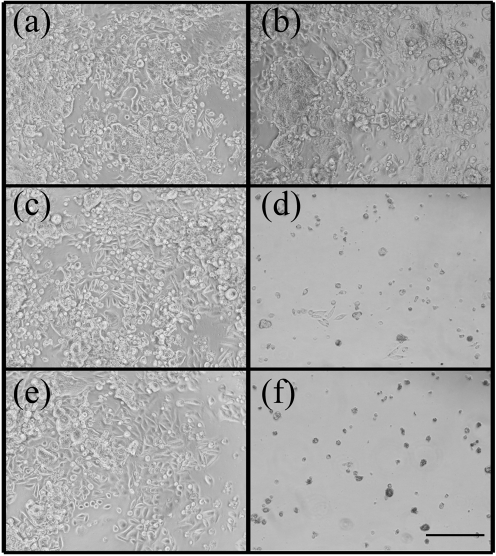
Micrographs of human SW480 colon cancer cells following 5 days of incubation with the different fatty acid treatments. (a, b) Linoleic acid control (extracted from broth); (a) 5 and (b) 20 μg (ml medium)^−1^. (c, d) *E. coli* (pEV01) fermented oils containing (c) 5 and (d) 20 μg (ml medium)^−1^ *t*10, *c*12 CLA. (e, f) *L. lactis* (pEV01) fermented oils containing (e) 5 and (f) 20 μg (ml medium)^−1^ *t*10, *c*12 CLA. All panels are ×100 magnification; bar, ** μm.

Microscopic examination of the SW480 colon cancer cells following 5 days of incubation with the various fatty acids treatments (Fig. 4) confirmed the results from the MTS cell viability assay (Fig. 3). SW480 cells incubated with 20 μg linoleic acid ml^−1^ were largely unaffected, in terms of either cell numbers or morphology (Fig. 4a, b[Fig f4]). In contrast, in the samples incubated with 20 μg ml^−1^ *L. lactis* and *E. coli* (pEV01) fermented oils, there was a noticeable reduction in cell numbers presenting a viable epithelial-like morphology. Cells undergoing cell death and associated morphological changes were also evident (Fig. 4c–f[Fig f4]).

An interesting observation was the increased inhibition of SW480 cell numbers in the samples treated with the *L. lactis* (pEV01)-generated fermented oil, compared with the samples treated with the same concentration of pure CLA standard or *E. coli* (pEV01) fermented oil. The added anti-proliferative effect of lactococcal-derived oil compared with that from *E. coli* may be due to other hydrophobic constituents which were co-extracted with the fatty acids. These putative constituents do not appear to have anti-proliferative activity on their own, given that the vector controls did not exhibit significant anti-proliferative activity. It is possible that such hydrophobic constituents act synergistically with the *t*10, *c*12 CLA isomer to give the observed increased killing with *L. lactis*-derived oils.

The *t*10, *c*12 CLA isomer has been shown to inhibit growth and induce apoptosis in a number of cell lines, such as HT-29 human colon cancer cells (Cho *et al.*, 2005, 2006), Caco-2 colon cancer cells (Kim *et al.*, 2002b), human colorectal cancer (CRC) cells (Lee *et al.*, 2006), dRLh-84 rat hepatoma cells (Yamasaki *et al.*, 2005) and the PC-3 human prostatic carcinoma cell line (Ochoa *et al.*, 2004), while similar effects have not been reported for the *c*9, *t*11 CLA isomer at the same concentrations.

Both *c*9, *t*11 CLA and *t*10, *c*12 CLA have been shown to inhibit growth of MCF-7 cells (Scultz *et al*., 1992; O'Shea *et al*., 1999). The human SW480 colon cancer cell line was chosen in the present study, as it has previously been shown to be efficient as an *in vitro* cancer model (Miller *et al.*, 2001, 2002, 2003). In the study of Miller *et al.* (2002), the *t*10, *c*12 CLA isomer was the most potent isomer examined, reducing cell viability by 47–61 % at a concentration of 16 μg ml^−1^, compared with a 40–52 % reduction by *c*9, *t*11 CLA at the same concentration. The same concentration of *t*10, *c*12 CLA reduces viability by 50–60 % in both SW480 and the MCF-7 breast cancer cell line following 4 days of incubation (Miller *et al.*, 2001). In contrast, 16 μg linoleic acid ml^−1^ increases viability of SW480 cells by 23 % (Miller *et al.*, 2001). Linoleic acid, however, has also been shown to inhibit growth of SW480 cells at higher concentrations. Incubation with 42.8 μg ml^−1^ (152.5 μM) linoleic acid reduces viability by 40 % after 3 days of incubation (Miller *et al.*, 2003).

In order to be a valuable therapeutic agent for cancer, CLA or any other anti-proliferative agent must demonstrate selective growth inhibition of cancer cells. A number of studies have presented evidence supporting such a claim for CLA for a range of tissue types. No cytotoxic or apoptotic effect of CLA (as a blend of isomers and specific isomers) has been found on human osteoblast-like cells (SaOS2 and MG63) in culture at a concentration range of 0–50 μM (Cusack *et al.*, 2005). Similarly, neither apoptosis nor any of the apoptosis regulatory proteins was affected by CLA in normal mammary gland alveoli or terminal end buds, whereas CLA inhibited the formation of pre-malignant lesions of rat mammary gland by ∼50 % and significantly increased apoptosis, reducing the expression of bcl-2 in these lesions (Ip *et al.*, 2000). Yamasaki *et al.* (2002) have demonstrated a decrease in viable cell numbers of normal rat RLN-10 hepatocytes by the *t*10, *c*12 CLA isomer only at 25 μM, compared with rat hepatoma dRLh-84 cells, which are much more sensitive to *t*10, *c*12 CLA: less than 25 % viable cells persisted following treatment with 1 μM of this isomer.

## CONCLUSIONS

In this study, we have heterologously expressed linoleic acid isomerase in *L. lactis* and *E. coli*, and in so doing generated cultures which can produce *t*10, *c*12 CLA. Moreover, this is believed to be the first instance in which the CLA produced by recombinant strains has been shown to exhibit an anti-proliferative effect. These results demonstrate the potential for using recombinant strains to deliver bioactive CLAs to (or produce within) the mammalian intestine, where they exert an anti-proliferative activity.

## Figures and Tables

**Table 1. t1:** Fatty acid composition of the various fatty acid treatments (% of total fatty acids in samples) Values represent the mean of triplicate analyses±sd.

**Fatty acid treatment**	**Fatty acid composition (percentage of total fatty acids)**					
	**C16 : 0**	**C18 : 0**	**C18 : 1 *c*9**	**C18 : 2 *c*9, *c*12**	**C18 : 2 *t*10, *c*12 CLA**	**C18 : 2 *t*9, *t*11 CLA**
Control linoleic acid	0.7±0.06	1.2±0.15	5.4±0.06	92.7±0.2	−	−
Pure *t*10, *c*12 CLA	−	−	−	−	100	−
*E. coli* (pEV01) fermented oils	4.2±0.0	1.6±0.07	6.2±0.0	49.2±0.07	37.3±0.28	1.6±0.42
*L. lactis* (pEV01) fermented oils	1.1±0.06	1.8±1.5	4.3±3.29	53.7±1.8	39.0±1.5	−
*E. coli* (pNZ44) vector control fermented oils	4.0±0.28	1.2±0.07	4.8±0.14	90.1±0.49	−	−
*L. lactis* (pNZ44) vector control fermented oils	0.8±0.07	0.9±0.07	4.9±0.07	93.3±0.07	−	−

## Abbreviations

- CLA, conjugated linoleic acid
- *c*9, *t*11 CLA, *cis-*9, *trans-*11 CLA
- *t*10, *c*12 CLA, *trans-*10, *cis-*12 conjugated linoleic acid
- PAI, *P. acnes* isomerase
